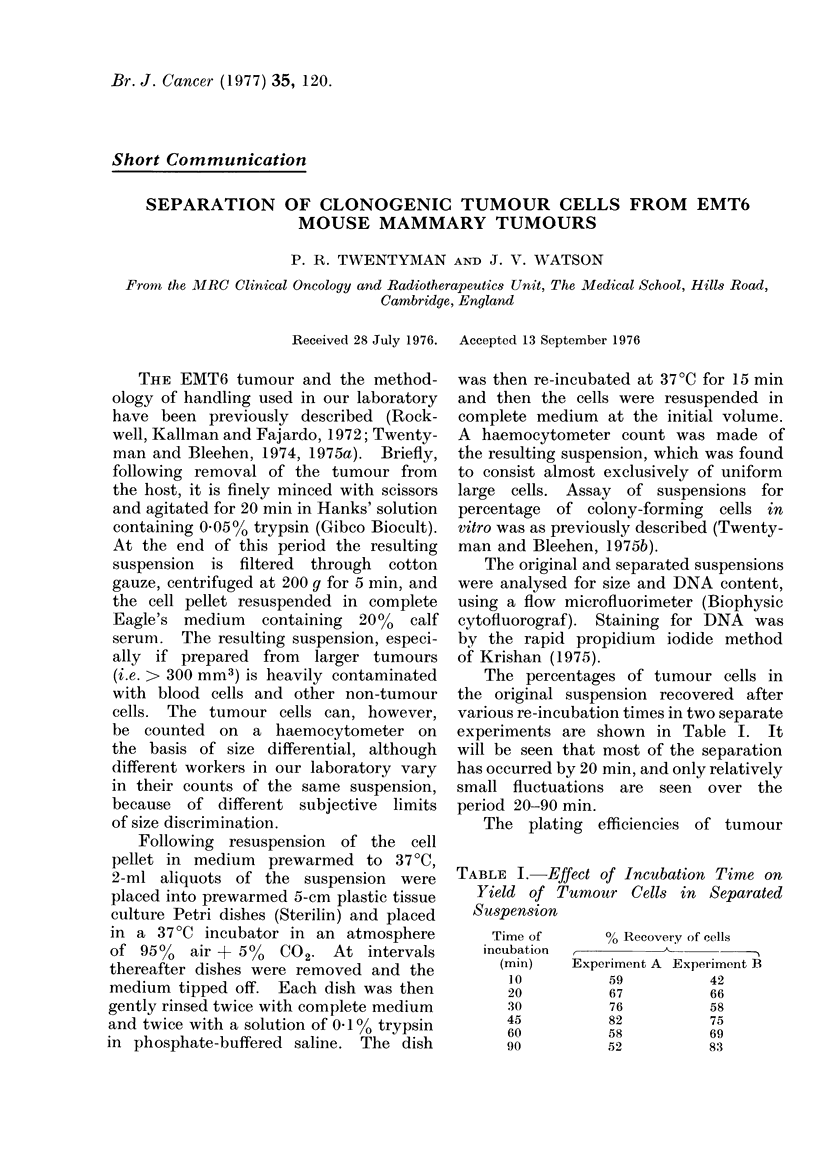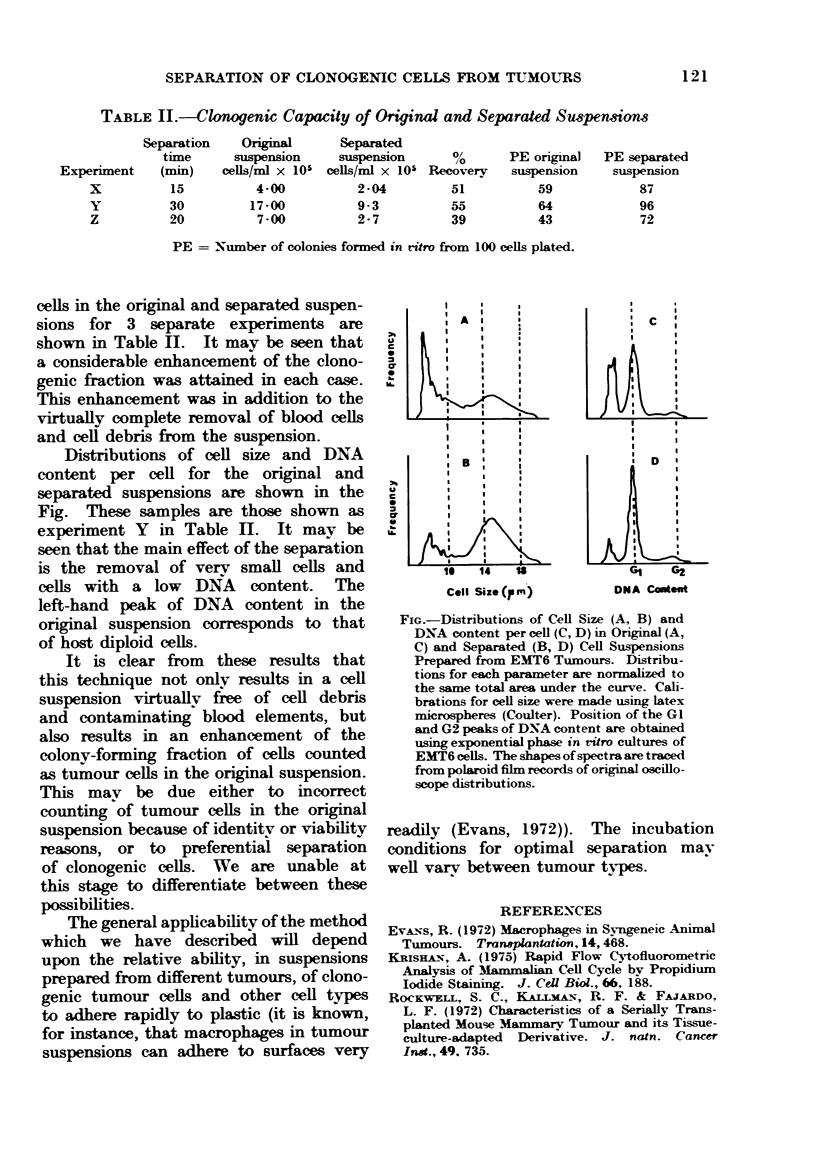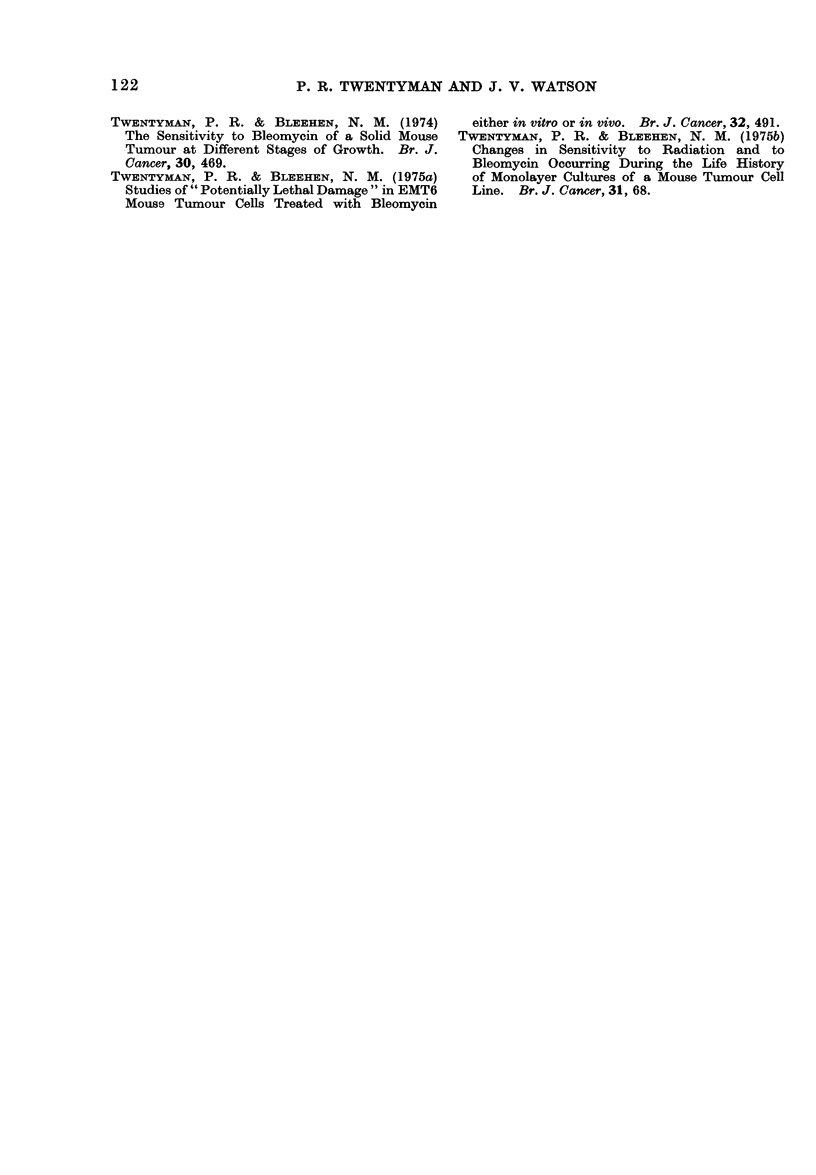# Separation of clonogenic tumour cells from EMT6 mouse mammary tumours.

**DOI:** 10.1038/bjc.1977.12

**Published:** 1977-01

**Authors:** P. R. Twentyman, J. V. Watson


*Br. J. Cancer* (1977) **35**, 120.

***Short Communication***

### SEPARATION OF CLONOGENIC TUMOUR CELLS FROM EMT6 MOUSE MAMMARY TUMOURS

P. R. TWENTYMAN AND J. V. WATSON

*From the MRC Clinical Oncology and Radiotherapeutics Unit, The Medical School, Hills Road, Cambridge, England*

Received 28 July 1976. Accepted 13 September 1976

THE EMT6 tumour and the methodology of handling used in our laboratory have been previously described (Rockwell, Kallman and Fajardo, 1972; Twentyman and Bleehen, 1974, 1975*a*). Briefly, following removal of the tumour from the host, it is finely minced with scissors and agitated for 20 min in Hanks' solution containing 0·05% trypsin (Gibco Biocult). At the end of this period the resulting suspension is filtered through cotton gauze, centrifuged at 200 *g* for 5 min, and the cell pellet resuspended in complete Eagle's medium containing 20% calf serum. The resulting suspension, especially if prepared from larger tumours (*i.e.* > 300 mm$^3$) is heavily contaminated with blood cells and other non-tumour cells. The tumour cells can, however, be counted on a haemocytometer on the basis of size differential, although different workers in our laboratory vary in their counts of the same suspension, because of different subjective limits of size discrimination.

Following resuspension of the cell pellet in medium prewarmed to 37°C, 2-ml aliquots of the suspension were placed into prewarmed 5-cm plastic tissue culture Petri dishes (Sterilin) and placed in a 37°C incubator in an atmosphere of 95% air + 5% $CO_2$. At intervals thereafter dishes were removed and the medium tipped off. Each dish was then gently rinsed twice with complete medium and twice with a solution of 0·1% trypsin in phosphate-buffered saline. The dish was then re-incubated at 37°C for 15 min and then the cells were resuspended in complete medium at the initial volume. A haemocytometer count was made of the resulting suspension, which was found to consist almost exclusively of uniform large cells. Assay of suspensions for percentage of colony-forming cells *in vitro* was as previously described (Twentyman and Bleehen, 1975*b*).

The original and separated suspensions were analysed for size and DNA content, using a flow microfluorimeter (Biophysic cytofluorograf). Staining for DNA was by the rapid propidium iodide method of Krishan (1975).

The percentages of tumour cells in the original suspension recovered after various re-incubation times in two separate experiments are shown in Table I. It will be seen that most of the separation has occurred by 20 min, and only relatively small fluctuations are seen over the period 20–90 min.

The plating efficiencies of tumour

TABLE I.—*Effect of Incubation Time on Yield of Tumour Cells in Separated Suspension*

| Time of incubation (min) | % Recovery of cells | |
|---|---|---|
| | Experiment A | Experiment B |
| 10 | 59 | 42 |
| 20 | 67 | 66 |
| 30 | 76 | 58 |
| 45 | 82 | 75 |
| 60 | 58 | 69 |
| 90 | 52 | 83 |

TABLE II.—*Clonogenic Capacity of Original and Separated Suspensions*

| Experiment | Separation time (min) | Original suspension cells/ml × $10^5$ | Separated suspension cells/ml × $10^5$ | % Recovery | PE original suspension | PE separated suspension |
|---|---|---|---|---|---|---|
| X | 15 | 4·00 | 2·04 | 51 | 59 | 87 |
| Y | 30 | 17·00 | 9·3 | 55 | 64 | 96 |
| Z | 20 | 7·00 | 2·7 | 39 | 43 | 72 |

PE = Number of colonies formed *in vitro* from 100 cells plated.

cells in the original and separated suspensions for 3 separate experiments are shown in Table II. It may be seen that a considerable enhancement of the clonogenic fraction was attained in each case. This enhancement was in addition to the virtually complete removal of blood cells and cell debris from the suspension.

Distributions of cell size and DNA content per cell for the original and separated suspensions are shown in the Fig. These samples are those shown as experiment Y in Table II. It may be seen that the main effect of the separation is the removal of very small cells and cells with a low DNA content. The left-hand peak of DNA content in the original suspension corresponds to that of host diploid cells.

It is clear from these results that this technique not only results in a cell suspension virtually free of cell debris and contaminating blood elements, but also results in an enhancement of the colony-forming fraction of cells counted as tumour cells in the original suspension. This may be due either to incorrect counting of tumour cells in the original suspension because of identity or viability reasons, or to preferential separation of clonogenic cells. We are unable at this stage to differentiate between these possibilities.

The general applicability of the method which we have described will depend upon the relative ability, in suspensions prepared from different tumours, of clonogenic tumour cells and other cell types to adhere rapidly to plastic (it is known, for instance, that macrophages in tumour suspensions can adhere to surfaces very readily (Evans, 1972)). The incubation conditions for optimal separation may well vary between tumour types.

FIG.—Distributions of Cell Size (A, B) and DNA content per cell (C, D) in Original (A, C) and Separated (B, D) Cell Suspensions Prepared from EMT6 Tumours. Distributions for each parameter are normalized to the same total area under the curve. Calibrations for cell size were made using latex microspheres (Coulter). Position of the G1 and G2 peaks of DNA content are obtained using exponential phase *in vitro* cultures of EMT6 cells. The shapes of spectra are traced from polaroid film records of original oscilloscope distributions.

### REFERENCES

EVANS, R. (1972) Macrophages in Syngeneic Animal Tumours. *Transplantation*, **14**, 468.

KRISHAN, A. (1975) Rapid Flow Cytofluorometric Analysis of Mammalian Cell Cycle by Propidium Iodide Staining. *J. Cell Biol.*, **66**, 188.

ROCKWELL, S. C., KALLMAN, R. F. & FAJARDO, L. F. (1972) Characteristics of a Serially Transplanted Mouse Mammary Tumour and its Tissue-culture-adapted Derivative. *J. natn. Cancer Inst.*, **49**, 735.

TWENTYMAN, P. R. & BLEEHEN, N. M. (1974) The Sensitivity to Bleomycin of a Solid Mouse Tumour at Different Stages of Growth. *Br. J. Cancer*, **30**, 469.

TWENTYMAN, P. R. & BLEEHEN, N. M. (1975*a*) Studies of " Potentially Lethal Damage " in EMT6 Mouse Tumour Cells Treated with Bleomycin either *in vitro* or *in vivo*. *Br. J. Cancer*, **32**, 491.

TWENTYMAN, P. R. & BLEEHEN, N. M. (1975*b*) Changes in Sensitivity to Radiation and to Bleomycin Occurring During the Life History of Monolayer Cultures of a Mouse Tumour Cell Line. *Br. J. Cancer*, **31**, 68.